# Capacity assessment of selected health care facilities for the pilot implementation of Package for Essential Non-communicable Diseases (PEN) intervention in Ghana

**DOI:** 10.11604/pamj.supp.2016.25.1.6252

**Published:** 2016-10-01

**Authors:** Kofi Mensah Nyarko, Donne Kofi Ameme, Dennis Ocansey, Efua Commeh, Mehitabel Tori Markwei, Sally-Ann Ohene

**Affiliations:** 1 Ghana Field Epidemiology and Laboratory Training Programme, School of Public Health, University of Ghana, Box LG 13, Legon, Accra, Ghana; 2 Disease Control and Prevention Department, Ghana Health Service, Box KB 493, Korle-Bu, Accra, Ghana; 3 Yale University, New Haven, USA; 4 World Health Organization, Ghana Country Office Box MB 42, Accra, Ghana

**Keywords:** Capacity assessment, Non-Communicable Diseases, WHO-PEN, Ghana

## Abstract

**Introduction:**

Non-communicable diseases (NCDs) continue to pose threats to human health and development worldwide. Though preventable, NCDs kill more people annually than all other diseases combined. The four major NCDs namely cardiovascular diseases, chronic respiratory diseases, diabetes and cancers share common modifiable risk factors. In order to prevent and control NCDs, Ghana has adopted the World Health Organisation Package for Essential NCD (WHO-PEN) intervention, to be piloted in selected districts before a nationwide scale-up. We assessed the capacity of these facilities for the implementation of the WHO-PEN pilot.

**Methods:**

We conducted a cross-sectional health facility-based survey using a multistage sampling technique. We collected data on human resource, equipment, service utilization, medicines availability and health financing through interviews and observation. Descriptive data analysis was performed and expressed in frequencies and relative frequencies.

**Results:**

In all, 23 health facilities comprising two regional hospitals, three district hospitals, nine health centres and nine Community-based Health Planning and Services (CHPS) compounds from three regions were surveyed. All the hospitals had medical officers whilst 4 (44.4%) of the health centres had physician assistants. Health financing is mainly by the National Health Insurance Scheme (NHIS). None of the health facilities had spacers and only one health centre had oxygen cylinder, glucometer and nebulizer.

**Conclusion:**

Gaps exist in the human resource capacity and service delivery at the primary care levels, the focus of WHO-PEN intervention. Adequately equipping the primary health care level with trained health workers, basic equipment, medications and diagnostics will optimize the performance of WHO-PEN intervention when implemented.

## Introduction

Non-communicable diseases (NCDs) continue to be a major public health problem worldwide posing threats to human health and development. They are the leading cause of death worldwide killing more than 36 million people each year [1, 2]. The four major NCDs namely cardiovascular diseases (CVDs), chronic respiratory diseases (CRD), diabetes and cancers kill more people each year than all other diseases combined [3]. The bulk of the mortality burden falls disproportionately on low and middle-income countries where nearly 80% of all NCDs deaths and 90% of all deaths before age 60 years attributed to NCDS occur [1]. These premature deaths deprive nations of economically active population. The economic impact is therefore substantially greater for low and middle-income countries (LMICs) because working-age adult accounts for the bulk of the NCD burden. In sub-Saharan Africa, NCDs are projected to be the commonest cause of death by 2030 [1, 3–5]. In Ghana, NCDs contribute significantly to the morbidity and mortality. Prevalence of hypertension in adults is between 24 % and 48% [6] whilst prevalence of diabetes in major cities is between 6%-9% [7, 8]. Also, NCDs kill an estimated 78,000 persons in Ghana annually, representing 354 deaths per 100,000 population [1].

WHO estimates that up to 80% of NCDs are preventable through lifestyle changes [3]. The four most common NCDs (CVD, cancers, CRD, and diabetes) share modifiable risk factors namely tobacco use, physical inactivity, harmful use of alcohol and unhealthy diets [1]. Effective primary prevention strategies exist and require risk assessment and management. Though risk assessment and clinical decision support tools are readily used in high-income settings, these are hardly replicable in low resource settings. The WHO-PEN intervention has been developed as a risk management package for NCDs to facilitate multiple risk factor assessment and treatment in low resource settings [9].

The WHO-PEN intervention is a prioritized set of cost-effective interventions that provides clinical decision support for assessment and management of NCDs at the primary care level in low resource settings [9]. It is designed to use cost-effective interventions for early detection, prevention and treatment of the major NCDs namely heart attacks and strokes, diabetes, cancer, renal diseases and asthma. The package uses simple algorithms to stratify patients’ risk status based on age, clinical history, comorbidities and blood pressure for care. Adaptation of WHO-PEN intervention for primary healthcare level in Ghana has been suggested [10] with the expectation of reducing hospital admissions related to NCDs.

Ghana has therefore adopted this tool with a strategy of piloting it in selected health facilities and ultimately scaling up to cover the whole country. Since the successful implementation of this intervention will largely depend on the readiness of the health facilities, there is the need to determine the capacity of the health facilities in order to identify existing and potential gaps that may hamper the smooth deployment of the package. Our study therefore responded to this need with the objective of assessing human resource capacity, equipment, service utilization, medicines availability and health financing.

## Methods

### Design and setting

We conducted a cross-sectional health facility-based survey from 9th June to 28th June 2013 in three districts in Ghana, a West African country with a population of 24,658,823 [11]. Ghana covers a land size of 238,533 square kilometres with a population density of 103 per square kilometre. It is bounded to the north by Burkina Faso, east by Togo and west by Ivory Coast. The national capital, Accra, is located in the Greater Accra region. Administratively, the country is organized into regions, which are sub divided into districts, municipalities, or metropolitan areas based on their populations. At present there are 10 regions, 216 metropolis, municipalities and districts. The ten regions of Ghana were zoned into three namely southern, middle and northern zones based on their geographical location. In each of the zones, one region was randomly selected. Three regions namely, Eastern Region, BrongAhafo Region and Northern Region were randomly selected to represent the southern, middle and northern zones respectively. In each of the selected regions, one district was randomly selected as the study site as follows: West Gonja District in the Northern region representing the Northern zone, Dormaa District in the BrongAhafo region representing the middle zone and Upper Manya district in the Eastern region representing the Southern zone of Ghana.

### Selection of health facilities

The health system is organized at different levels from the lowest level of care called the Community-based Health Planning and Services (CHPS) compound which are manned by Community health nurses, through health centres which are manned by medical assistants, then district hospitals which are manned by medical officers and provide general medical services, regional hospitals which provide some level of specialized services and the teaching hospitals. Each region has one regional hospital. There are three teaching hospitals, one in each zone. Approximately 58% of the population live within 30 minutes of a health facility with urban households having better geographical access (78.5%) compared to their rural counterparts (42.3%) [12].

In each of the selected regions, the regional hospital was purposively selected to reflect facilities with high caseload, high cadre of personnel and advanced case-management skills. In each of the selected districts, the district hospital was selected in addition to three health centres and three Community-based Health and Planning Services (CHPS) zones in order to reflect the referral system. The health centres and the CHPS zones were purposively selected based on their distances from the district hospital which served as the main referral centre in the district: one near and one far from the hospital and a third one in between these two.

### Data collection

The assessment had approval of the authorities of the Ghana Health Service as part of the responsibilities of the NCD Control Programme. Permission was also sought from the respective Regional Health Directors, District Directors of Health Services as well as the medical directors and heads of the health facilities. All of them willingly agreed for their facilities to be included for data collection. Trained health workers collected data from each of the selected districts through a combination of self-administered questionnaires and interviews. The authorities of the facilities and key personnel who could provide information were trained and given self-administered questionnaires to obtain information on human resource, infrastructure and equipment, service utilization, referrals, medicines and health financing. The survey team reviewed the completed questionnaires with the respondents to ensure that the questionnaires were properly filled. Where necessary, the responses to the questions were validated by observation and inspection of the facilities. A rapid assessment tool for primary healthcare facility capacity assessment for NCDs was adapted and used for data collection. All the facilities, except one regional hospital returned the completed questionnaire.

### Data processing and analysis

We performed descriptive statistical analysis and expressed categorical variables as frequencies and relative frequencies. Data was entered cleaned and analysed using Epi Info version 7.

## Results

In all, 24 health facilities from the three regions were surveyed. These include two regional hospitals, three district hospitals, nine health centres and nine CHPS. Table 1 shows a breakdown of the health facilities included in the survey by ownership and setting. Majority 21 (92.0%) of the health facilities were owned by the government of Ghana. Two were owned by not-for-profit faith-based organisations but supported by government and therefore considered quasi-governmental.

**Table 1:** Characteristics of the surveyed health facilities, Ghana, 2013

Characteristics	Facility Type			
	CHPS	Health Centre	District Hospital	Regional Hospital
	n = 9	n = 9	n = 3	n = 2
**Ownership**				
Public	9	8	1	2
Private	0	0	1	0
Quasi- government	0	1	1	0
**Setting**				
Rural	9	7	0	0
Urban	0	2	3	2

### Human resource

All the CHPS compounds have at least a trained nurse or health assistant manning them. At the health centres however, four (44.4%) out of the nine had medical assistants. Trained nurses, mostly midwives, were managing the rest. All the district hospitals had at least one medical officer in charge. The regional and teaching hospitals had physician specialists and other specialist doctors delivering care. Other categories of staff working in all the primary health care setting included laboratory technicians, pharmacy assistants or dispensing technicians, and community and public health nurses. In 5 out of the 9 (56%) health centres, there were health promoters who were involved in giving health education in the communities through house-to-house and other engagements such as school and church programmes.

### Equipment and diagnostics

Basic equipment for managing NCDs were not readily available in most of the health facilities particularly the primary health care level. None of the CHPS centres had functional glucometers, oxygen cylinders or nebulizers and only 1 out of the 9 (11%) health centres had these equipment. There was no functional spacer in any of the health facilities (Table 2). All the facilities had functional blood pressure measuring devices (BPMD) as well as weighing scales. Diagnostic tests were virtually non-existent in the CHPS compounds and the health centres. With the exception of serum troponin levels for ischaemic heart disease, the regional hospitals have all the basic diagnostic tests (Table 3).

**Table 2:** Availability of basic equipment in surveyed health facilities, Ghana, 2013

Equipment	Health Facility Type			
	CHPS	Health Centre	District Hospital	Regional Hospital
	n (%)	n (%)	n (%)	n (%)
Functional oxygen cylinder	0(0.0)	1(11.1)	3(100.0)	2(100.0)
Functional BPMD	9(100.0)	9(100.0)	3(100.0)	2(100.0)
Functional weighing scale	9(100.0)	9(100.0)	3(100.0)	2(100.0)
Functional Glucometer	0(0.0)	1(11.1)	3(100.0)	2(100.0)
Functional Nebulizer	0(0.0)	1(11.1)	3(100.0)	2(100.0)
Functional Spacer	00.0)	0(0.0)	0(0.0)	0(0.0)
Functional Peak Flow Meter	0(0.0)	0(0.0)	1(33.3)	2(100.0)
Functional Pulse oxymeter	0(0.0)	1(11.1)	3(100.0)	2(100.0)
Functional Health education materials	1(11.1)	5(55.6)	2(66.7)	2(100.0)
Functional tape measures	6(66.7)	9(100.0)	3(100.0)	2(100.0)
Functional ECG	0(0.0)	0(0.0)	2(66.7)	2(100.0)
Functional Stethoscopes	9(100.0)	9(100.0)	3(100.0)	2(100.0)
Functional Thermometers	9(100.0)	9(100.0)	3(100.0)	2(100.0)

**Table 3:** Availability of basic diagnostic tests in surveyed health facilities, Ghana, 2013

Diagnostic Test	Health Facility Type			
	CHPS	Health Centre	District Hospital	Regional Hospital
	n(%)	n(%)	n(%)	n(%)
Urine albumin/protein	0(0.0)	3(33.3)	3(100.0)	2(100.0)
Urine glucose	0(0.0)	3(33.3)	3(100.0)	2(100.0)
Urine ketones	0(0.0)	1(11.1)	3(100.0)	2(100.0)
Blood glucose	0(0.0)	1(11.1)	3(100.0)	2(100.0)
SerumTroponin	0(0.0)	0(0.0)	0(0.0)	0(0.0)
Blood cholesterol	0(0.0)	0(0.0)	2(66.7)	2(100.0)
Serum creatinine	0(0.00	0(0.0)	2(66.7)	2(100.0)

The two regional hospitals had almost all the essential medicines for managing NCDs (Table 4). The CHPS compounds and the health centres lacked most of the drugs. Glucose injectables were lacking in some of the health centres and the CHPS compounds. None of the CHPS compounds had salbutamol inhaler available. In the case of health centres, only four of them had salbutamol inhaler.

**Table 4:** Availability of selected essential medicines in surveyed facilities, Ghana, 2013

Selected Essential Medicine	Health Facility Type			
	CHPS	Health Centre	District Hospital	Regional Hospital
	n(%)	n(%)	n(%)	n(%)
Adrenaline injection	2(22.2)	4(44.4)	3(100.0)	2(100.0)
Aspirin	9(100.0)	5(55.5)	3(100.0)	2(100.0)
Atenolol/Beta blockers	0(0.0)	1(11.1)	3(100.0)	2(100.0)
Beclomethasone inhaler	0(0.0)	0(0.0)	2(66.7)	2(100.0)
Bendrofluazide	0(0.0)	1(11.1)	3(100.0)	2(100.0)
Benzathine Penicillin	5(55.6)	6(66.7)	3(100.0)	2(100.0)
Enalapril/ Lisinopril	0(0.0)	1(11.1)	3(100.0)	2(100.0)
Erythromycin	9(100.0)	3(33.3)	3(100.0)	2(100.0)
Furosemide	0(0.0)	3(33.3)	3(100.0)	2(100.0)
Glibenclamide	0(0.0)	1(11.1)	3(100.0)	2(100.0)
Hydrocortisone (injection)	8(88.9)	8(88.9)	3(100.0)	2(100.0)
Insulin (long acting)	0(0.0)	0(0.0)	2(66.7)	2(100.0)
Insulin (soluble)	0(0.0)	0(0.0)	3(100.0)	2(100.0)
Ipratropium bromide	0(0.0)	0(0.0)	0(0.0)	1(50.0)
Isosorbidedinitrate	0(0.0)	0(0.0)	2(66.7)	2(100.0)
Statins	0(0.0)	0(0.0)	1(33.3)	2(100.0)
Metformin	0(0.0)	1(11.1)	3(100.0)	2(100.0)
Calcium channel blockers	6(66.7)	4(44.4)	3(100.0)	2(100.0)
Sodium chloride infusion	7(77.8)	9(100.0)	3(100.0)	2(100.0)
Phenoxymethyl Penicillin	0(0.0)	4(44.4)	2(66.7)	2(100.0)
Prednisolone	1(11.1)	3(33.3)	3(100.0)	2(100.0)
Salbutamol inhaler	0(0.0)	4(44.4)	3(100.0)	2(100.0)
Salbutamol tablet	3(33.3)	7(77.8)	3(100.0)	2(100.0)
Salbutamol injection	0(0.0)	0(0.0)	2(66.7)	2(100.0)
Paracetamol	9(100.0)	9(100.0)	3(100.0)	2(100.0)
Ibuprofen	9(100.0)	9(100.0)	3(100.0)	2(100.0)
Codeine	0(0.0)	0(0.0)	1(33.3)	2(100.0)
Morphine (oral)	0(0.0)	0(0.0)	0(0.0)	2(100.0)
Morpine (injection)	0(0.0)	0(0.0)	1(33.3)	2(100.0)
Glyceryltrinitrate	0(0.0)	0(0.0)	0(0.0)	2(100.0)
Heparin	0(0.0)	0(0.0)	3(100.0)	2(100.0)
Amoxycillin	9(100.0)	9(100.0)	3(100.0)	2(100.0)
Cotrimoxazole	9(100.0)	9(100.0)	3(100.0)	2(100.0)
Promethazine injection	7(77.8)	7(77.8)	3(100.0)	2(100.0)
Glucose injectable	7(77.8)	8(88.9)	3(100.0)	2(100.0)

Available means always or available within the last six months prior to the assessment

### Service utilization and medical information system

All the facilities surveyed had medical registers where patients’ attendance records were documented. The patients’ folders were retrieved each time they visited the facilities using their unique folder identification numbers. None of the facilities had a registry (computerized version) of patients’ records.

Attendance of the patients to the facilities was largely by “walk–in” in all the facilities. A few 8 (34.8%) facilities use both ‘walk-in’ and ‘appointment’ system where patients call and book appointments for attendance. This appointment system usually works in the CHPS compounds where the health workers are in direct contact with the community members. Most of these appointments are not formalized and are based on the availability of the health worker. All the health facilities provided some education and counseling of patients on risk factors for NCDs. A few, 8 (34.8%) of the 24 health facilities were performing clinical breast examination.

All the lower health facilities were able to refer patients to a higher level. The lower facilities usually used other means of transporting patients apart from an ambulance. Some were able to arrange for ambulance to transport patients. Feedback on referred patients from the referral centres was a major challenge mentioned by all the primary health care facilities.

### Health financing

Ghana has a National Health Insurance Scheme (NHIS) as part of the health financing schemes and those who register by paying a premium are eligible to benefit under the scheme. All the 23 health facilities are accredited by the NHIS. Services and medications that are covered by the NHIS are therefore paid for through the NHIS except for individuals who do not have valid National Health Insurance cards.

## Discussion

The WHO-PEN is designed to deliver low-cost, high-impact interventions through the primary health care approach. This requires prioritization of resources geared towards adequately equipping the primary health care facilities and providing the required capacity to deliver care. From the results, the health facilities lacked the capacity required for the smooth implementation of the WHO-PEN intervention. Inadequate capacity of primary healthcare facilities to serve NCD-related health needs have been widely reported [2, 10, 13, 14]. The inadequate staffing was a major impediment. Since the successful functioning of the package will depend largely on human resource, the observation of inadequate resourcing of the facilities was a major setback. It brings to focus the need to empower non physician health workers to deliver NCD interventions consistent with their level of care as has been done elsewhere with satisfactory results [15, 16]. Though the package has been designed for low resource settings, certain prerequisites such as fair financing, trained personnel, essential equipment, diagnostics and medications are key to its implementation [9].

Despite the fact that some basic equipment were found in all the facilities, some vital equipment were also missing. Capacity of the health facilities was strong in the area of health financing. This is largely due to the NHIS, which covers treatment of most diseases Ghanaians are afflicted with [17]. With regards to health financing, it seems the NHIS caters for almost all the basic drugs required for the smooth implementation of the package, thus reducing the out-of-pocket expenditure on health. This contrasts with what was found in other LMICs [2]. Ghana’s NHIS therefore offers an opportunity to reduce the financial barriers that could have negatively affected the implementation of essential NCDs interventions at the primary care level.

Systems for managing patients’ information for continuity of care were inadequate in all the facilities surveyed. None of the facilities had a database of their patients that could facilitate follow up. Patients were referred when necessary, however feedback from the referral centres and adequate record on the patients were challenges. The records of the patients were not kept with sufficient care to enable tracking of their progress. This has effect on effective implementation of the WHO-PEN intervention. Since NCDs require long-term care and tracking of patients’ progress, having a good data management system in place would have been an added advantage.

The results also highlight the unavailability of essential medicines required for adequate management of NCDs at the various levels of care. The availability of the medicines reflected the capacity of the different levels of care. Since the different levels of health facilities have been mandated to cater for severity and complexity of diseases commensurate with their manpower expertise, the lower levels of care such as the CHPS compounds and the health centres did not have most of the medications available. The two regional hospitals had almost all the essential drugs available sometimes or always. The unavailability of glucose injectablesand salbutamol inhaler at the lower levels of care means that emergency situations such as hypoglycaemia and acute asthmatic attacks may not be managed as promptly as required. This has the potential for causing needless deaths.

Capacity of the health facilities was strong in the area of health promotion as all were providing some education of risk factors for NCDs and a few were providing screening for breast lesions through clinical breast examination. Though most of the lower levels of care did not show evidence of availability of health education materials, awareness creation was done through other means employed by the health promoters.

Although the study provides the opportunity for strengthening the baseline capacity of selected health facilities for prevention and management of NCDs, it is important to note that strong capacity does not necessarily imply optimal care. Also, the capacity of these facilities may change over time. Thus, there is the need for a follow-up and periodic assessment of the facilities and the impact of WHO-PEN intervention on NCDs control. The adoption of the WHO-PEN intervention will also set the stage for a more holistic approach to NCD control. It has been argued that health facility-based measures alone may not be enough to achieve the needed effects for priority NCDs [18]. There is therefore the need for sustained, comprehensive and multi-sectorial efforts beyond the confines of the health facilities [19, 20]. Prioritizing NCD control and investing resources to create awareness to address the existing and potential barriers to the implementation of the WHO-PEN is critical for a national scale-up. Integrating the WHO-PEN intervention with community wide health promotion activities therefore requires an urgent consideration.

## Conclusion

The preparedness of the health facilities for the implementation of WHO-PEN intervention is unsatisfactory. Apart from health financing, which seems to be uniform and somewhat adequate due to the NHIS, major gaps in the human resource capacity, availability of medications, diagnostics, equipment and medical information management system are likely to hamper the smooth implementation of the WHO-PEN intervention. Adequately addressing these gaps and other potential needs of the health facilities will optimize the implementation of the package.
